# The Mechanical Properties and Microstructure of Tailing Recycled Aggregate Concrete

**DOI:** 10.3390/ma17051058

**Published:** 2024-02-25

**Authors:** Fan Xu, Zhijun Li, Tao Li, Sheliang Wang

**Affiliations:** 1 School of Civil Engineering, Xi’an Technological University, Xi’an 710021, China; lzjsjh@aliyun.com; 2 Xi’an Key Laboratory of Civil Engineering Testing and Destruction Analysis on Military-Civil Dual Use Technology, Xi’an 710021, China; 3 College of Architecture Engineering, Huanghuai University, Zhumadian 463000, China; litao623114@126.com; 4 School of Civil Engineering, Xi’an University of Architecture and Technology, Xi’an 710055, China; sheliangw@163.com

**Keywords:** iron ore tailings, recycled coarse aggregate, compressive strength, microstructure

## Abstract

The aim of this study was to develop sustainable concrete by recycling concrete aggregates from steel waste and construction waste (iron ore tailings (IOTs) and recycled coarse aggregates (RCAs)) to replace silica sand and natural coarse aggregates. In experimental testing, the compressive strength, peak strain, elastic modulus, energy dissipated under compression, and compressive stress–strain curve were analyzed. Microscopically, scanning electron microscopy and energy-dispersive spectrometry were employed to investigate the microstructural characteristics of the interfacial transition zone (ITZ), and the results were compared with the ITZs of natural aggregate concrete and recycled aggregate concrete (RAC). In addition, the pore structure of concrete was determined by nuclear magnetic resonance. The results revealed that an appropriate IOT content can improve the ITZ and compactness of RAC, as well as optimize the mechanical and deformation properties of RAC. However, due to the presence of a smaller number of microcracks on the surface of IOT particles, excessive IOTs could reduce the integrity of the matrix structure and weaken the strength of concrete. According to the research, replacing silica sand with 30% IOTs led to a reduction in the porosity and microcracking which resulted in a much denser microstructure.

## 1. Introduction

As one of the important components of concrete, natural coarse aggregate (NCA) has a huge annual consumption, and its non-renewable characteristics make it urgent to find effective alternative materials [1]. At the same time, the construction industry in the 21st century is expected to produce a large amount of construction and demolition (c and d) waste, which is expected to reach 2.59 billion tons by 2030 and 3.4 billion tons by 2050 [2]. Thus, researchers have advocated for the reuse of waste concrete, so as to realize the engineering value of recycled coarse aggregate (RCA) repeatedly, minimize the ecological impacts caused by the massive exploitation of natural resources, and make important contributions to sustainable development.

RAC produced by RCA partially or completely replacing NCA has significant economic and environmental benefits. Existing studies show that compared with NCA, due to the residual cement matrix attached to the surface of the RCA and the microcracks generated by impacts during the production of RCA, RCA shows certain disadvantages such as low strength, high water absorption, and high porosity. This leads to the failure of RCA to completely replace NCA, thus limiting its many engineering applications [3]. Some studies have found that the physical and mechanical properties of RCA are inferior to those of NCA [4], and the direct use of RCA to make concrete will reduce its compressive strength, splitting strength, and workability [5,6]. Many studies have been published on the important effects of the ITZ properties on the mechanical properties of concrete. Liu et al. [7] reported that higher amounts of calcium hydroxide (Ca(OH)_2_) and ettringite (AFt) are present in the interfacial transition zone (ITZ) of concrete, which have a high porosity and cause weak interfaces in concrete. You et al. [8] analyzed the ITZ of recycled aggregate concrete (RAC) using the nanoindentation technique. They found that there are three types of ITZ inside RAC, and the indentation modulus of any ITZ is lower than that of the old and new pastes, which is the weak zone of RAC. The initial microcracks in RAC usually first appear in the ITZ during the destructive process [9,10]. Therefore, it is necessary to improve the performance of RAC by strengthening the weak regions of RCA. Chen et al. [11] used nano-SiO_2_ as a mineral admixture to improve the properties of the old ITZ and the new ITZ of RAC. He clarified the influence of nano-SiO_2_ content on the fluidity of cement paste sand and mechanical properties based on the tests of fluidity, mechanical properties, and microstructure. Liu et al. [12] studied the modification effects of graphene oxide on RAC, and the results showed that graphene oxide could fill the pores of RAC and reduce the number of holes. But, such techniques are technically demanding and expensive.

Many industrial by-products such as ground, granulated blast furnace slag, iron ore tailings (IOTs), metakaolin, silica powder, and fly ash have been used as mineral admixtures to improve the properties of concrete. Among the available mineral admixtures, IOTs, as industrial solid waste generated in the process of iron ore beneficiation, discharge more than 10 billion tons of tailings and waste rocks every year around the world [2]. IOTs have a wide range of potential applications in resource saving and solid waste utilization. IOTs are an industrial waste residue after separating the iron concentrate, which can cause environmental problems such as haze, sandstorms, and river pollution if discharged arbitrarily. The chemical compositions of IOTs are dominated by oxides of silicon, aluminum, iron, calcium, and magnesium, and the minerals in IOTs are mainly quartz and feldspar minerals. These are very similar in composition to silica sand minerals, providing a necessary basis for IOTs to become raw materials for building materials. Recent studies by the present authors have confirmed that IOTs can be used as an alternative to silica sand in preparing concrete. The present authors conducted a cube compressive strength test, splitting tensile test, and durability test, and evaluated the influence of IOTs on the transverse and axial strain of RAC using a digital image correlation system. The results showed that IOTs can improve the performance of RAC [13,14,15,16]. Using IOTs to enhance the performance of recycled concrete seems to be a good choice.

## 2. Research Significance

The use of IOTs in concrete will benefit the treatment of solid waste (IOTs and RCA), as well as the dilemma of the growing shortage of raw materials in the construction industry. Previous studies have mainly focused on the influence of IOTs on the macroscopic mechanical properties of conventional concrete, as well as the evolution process and law of material or structural damage [17,18,19], while few studies have focused on the impact of IOTs on the microstructure of concrete. Meanwhile, RAC has disadvantages such as low strength, unstable durability, and large shrinkage compared with natural aggregate concrete (NAC). It was crucial for this study to make up for the weak research foundation of concrete (TRAC) using industrial waste (IOTs and RCA). We systematically analyzed and studied the mechanical properties and microstructure of TRAC from both the macro- and micro-levels, determined the optimal amount of IOTs, and laid a theoretical foundation for engineering application.

For this purpose, in this study, we formulated TRAC, and analyzed the influence of IOT replacement on the deformation properties of concrete, such as the compressive strength, peak strain, elastic modulus, and energy dissipation capacity. In addition, to study the porous structure of TRAC, the current paper presents observations on the microstructure improvement in the concrete via the ITZ with a scanning electron microscope (SEM). Nuclear magnetic resonance (NMR) was used to test the porosity and pore size distribution. The effects and influence mechanism of the introduction of IOTs on the deformation properties of concrete were studied at mesoscopic scales, providing a reference for the application of TRAC.

## 3. Materials and Methods

### 3.1. Materials

#### 3.1.1. Cement and IOTs

**Figure 1:**
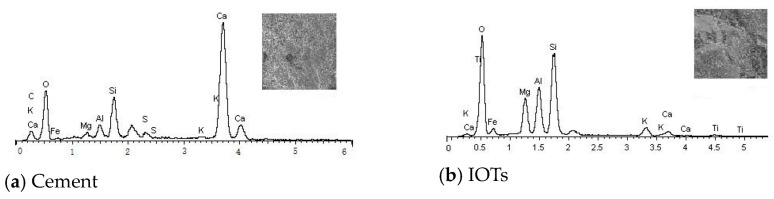
X-ray energy spectrum analysis (EDS) of cement and IOTs.

**Figure 2:**
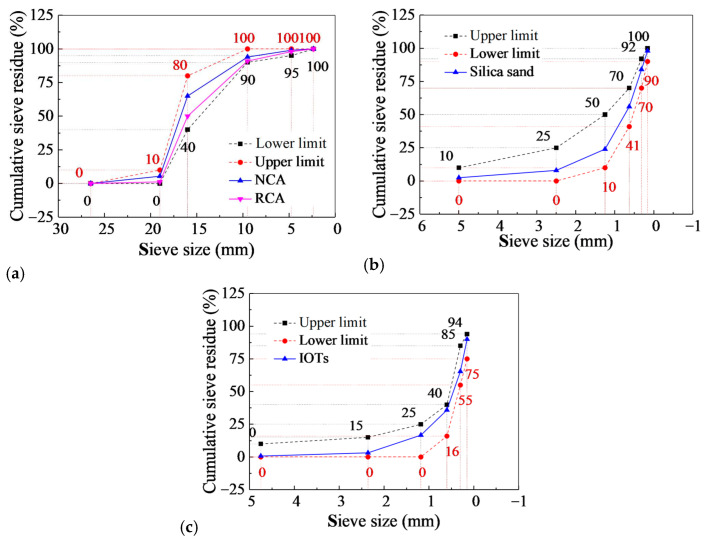
Particle gradation diagram: (**a**) coarse aggregate; (**b**) silica sand; (**c**) IOTs.

In this study, the cement was Ordinary Portland cement with a grade of 42.5, satisfying the Chinese national standard P.O.42.5R(II) [20]. The cement samples were analyzed by energy spectrum analysis (EDS). The main performance indicators and chemical elements of the cement are shown in Table 1 and Table 2. The selected IOTs were processed and collected from Shangluo Baoming Mining Co., Ltd., Shangluo, China, and used to replace natural sand in this study. The main performance indicators and chemical elements are listed in Figure 1, Table 1 and Table 3, and the gradation curve is shown in Figure 2c.

#### 3.1.2. Aggregates

The NCA and RCA used in this study both had a maximum particle size of 22 mm. The locally available crushed stones with a continuous gradation were used as the NCA. The RCA purchased from Xi’an, Shaanxi, was a common material in the current market. The selected RCA contained approximately 30% of the brick particles and was continuously graded. The purchased RCA was sieved, cleaned, dried, and bagged for test use. The main performance indicators of coarse aggregate are shown in Table 3, and the particle gradation is shown in Figure 2a. The silica sand was coarse sand acquired from the Weihe River in Xi’an, Shaanxi Province. The sieving curve of the silica sand obtained from the sieving test is shown in Figure 2b. The detailed performance indicators of the silica sand are given in Table 3.

#### 3.1.3. Water-Reducing Agent

The water-reducing agent used in this test was polycarboxylic acid high-performance water-reducing agent, and its main physical property indexes are shown in Table 4.

### 3.2. Mixing Proportion

According to previous publications [22,23,24,25], the impact on concrete was well within acceptable limits when the content of RCA was less than 30%. Therefore, the substitution ratio of RCA was fixed at 30% to facilitate research on the effect of IOTs on RAC mixtures.

The mix proportion designs were carried out according to the Chinese industry JGJ 55-2011 standard [26,27], and the mix proportions of the concrete mixes are reported in Table 5. The water/cement ratio was 0.4, and the sand ratio was 0.3.

The concrete mixtures were mixed with a horizontal double-shaft mixer. Firstly, the coarse aggregate and fine aggregate were dry-mixed for 1 min in the concrete mixer. Then, water and cement were added sequentially and stirred at low speed for 3 min. Finally, the mixture obtained was evenly casted into the well-oiled molds with different sizes and compacted using a vibrating table for 10 s to eliminate air bubbles during the casting. According to the national standard GB/T 50081 [28], demolding occurred after curing at room temperature for 24 h, curing at room temperature (24 ± 1 °C) and humidity (90 ± 5%) until use. The specimens with dimensions of 100 mm × 100 mm × 100 mm were produced for nuclear magnetic resonance (NMR) tests, and prismatic specimens with dimensions of 100 mm × 100 mm × 300 mm were used to evaluate the deformation properties. The average values of three specimens in each test were used as the results.

### 3.3. Test Method

#### 3.3.1. Deformation Property

Two types of specimens, 100 mm × 100 mm × 100 mm cubes and 100 mm × 100 mm × 300 mm prisms, were produced. The prisms were applied to test the axial compressive strength and stress–strain curve, whereas the cubes were applied to test pore structure. The compressive strength and stress–strain curve were evaluated by employing a WAW-1000 universal compression machine with a load capacity of 1000 kN in accordance with the Chinese national standard (GB/T50081-2002) [28]. According to Chinese standard GB/T50081-2002, the loading rate applied in the compressive strength test was 0.5 MPa/s.

#### 3.3.2. Micrograph Test

**Figure 3:**
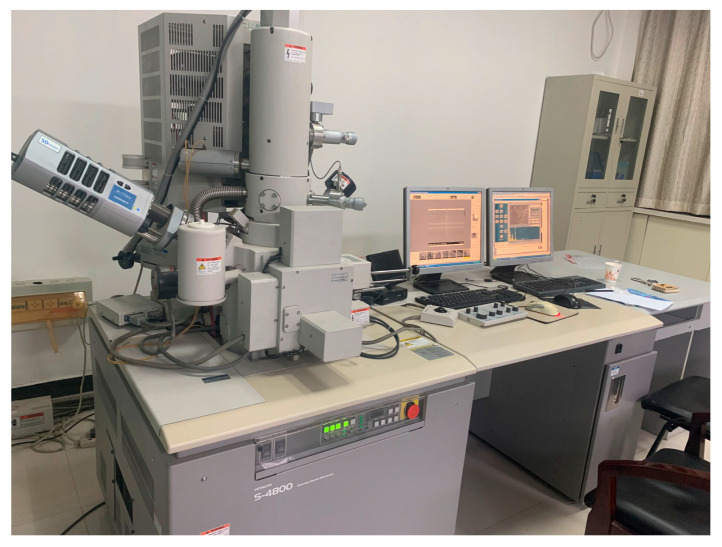
Hitachi S-4800 SEM.

All micrograph tests were conducted at the Materials Analysis and Testing Center of Chang’an University. The microstructure, textural characteristics, and morphological changes in RAC, NAC, and TRAC were obtained by means of a Hitachi S-4800 SEM (Figure 3). Each specimen was approximately 2 cm × 2 cm × 0.5 cm in size. Before the SEM observation, the specimen surface was sprayed with gold to ensure good electrical conductivity. After that, we placed specimens under vacuum for SEM observation. For further elemental composition, EDS line scans were performed using the EDS analyzer.

#### 3.3.3. Pore Structure

**Figure 4:**
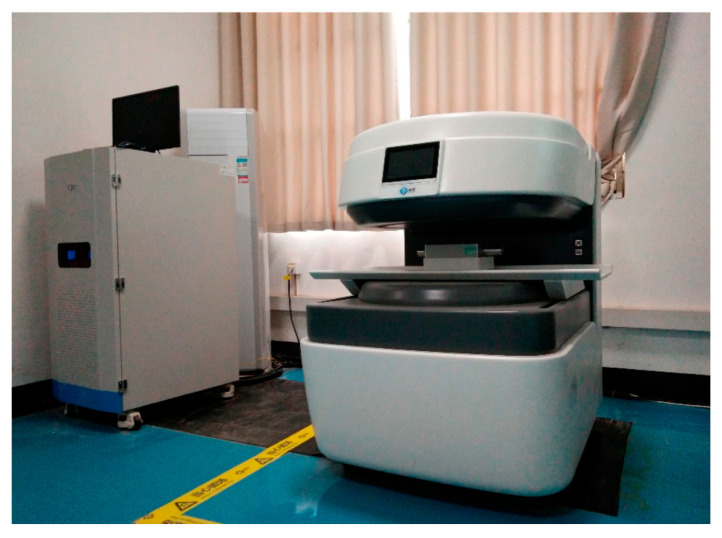
NMR spectrometer.

As is shown in Figure 4, the pore structure was measured by the low-field MacroMR12-150H-I NMR spectrometer from Newmai Analytical Instruments Co., Ltd. (Suzhou, China). The NMR spectrometer mainly consisted of a spectrometer system, RF unit, magnet cabinet and industrial computer. During the test, the magnetic field strength was set to 0.55 T and the H proton resonance frequency was 50–60 Hz.

The concrete specimens were cored by coring equipment. Before testing, the specimens were saturated with water under the vacuum environment for 24 h. After removal from water, the specimens were wiped to remove the surface moisture and wrapped with plastic wrap to avoid moisture loss. Finally, the specimens were put into the center space of the electromagnetic coil, and the CPMG (Carr–Purcell–Meiboom–Gill) pulse sequence was tested.

## 4. Result and Discussion

### 4.1. Deformation Property

#### 4.1.1. Axial Compressive Strength

**Figure 5:**
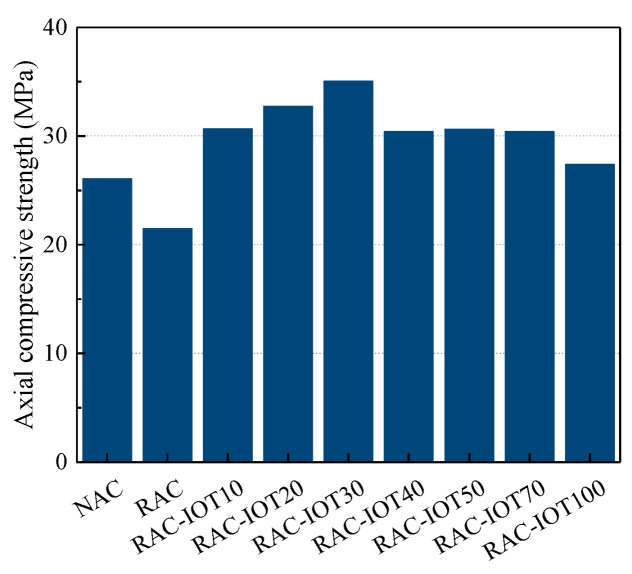
Effect of IOT content on the axial compressive strength of concrete.

Figure 5 shows the effect of IOT content on the axial compressive strength of specimens. The results showed that the axial compressive strength of NAC was 21.3% higher than that of RAC. The axial compressive strength values increased with the rise in IOT content when the content of IOTs was less than 30%. However, the strength decreased when the IOT content increased to more than 30%. Notably, the axial compressive strength of TRAC with various IOT contents was always higher than that of RAC, and some were even higher than that of NAC in this study. The axial compressive strength reached a maximum in the concrete with 30% IOTs. Therefore, the adverse effects caused by the use of RCA could be compensated for by incorporating IOTs into RAC, and the axial compressive strength of NAC can also be improved by incorporating IOTs into NAC; this fully illustrated the void filling performance of iron tailing particles.

The fact that excess IOTs led to a reduction in the axial compressive strength of the specimens can be explained with the following considerations: When IOTs became the main aggregates for concrete rather than the gap-filling component for optimum gradation, this resulted in an unreasonable aggregate gradation. The arch bridge effect occurred between particles with a small particle size (particles with similar sizes were staggered when the powder was stacked irregularly to form an arch bridge space and form larger pores) [29,30]. The initial damage within the concrete was increased by the large pores formed by the intergranular arch bridge effect (see Section 4.3), resulting in the strain concentration of concrete during the loading process. This was proved by the previous literature by the present authors (see reference [16] for reviews).

#### 4.1.2. Peak Strain

**Figure 6:**
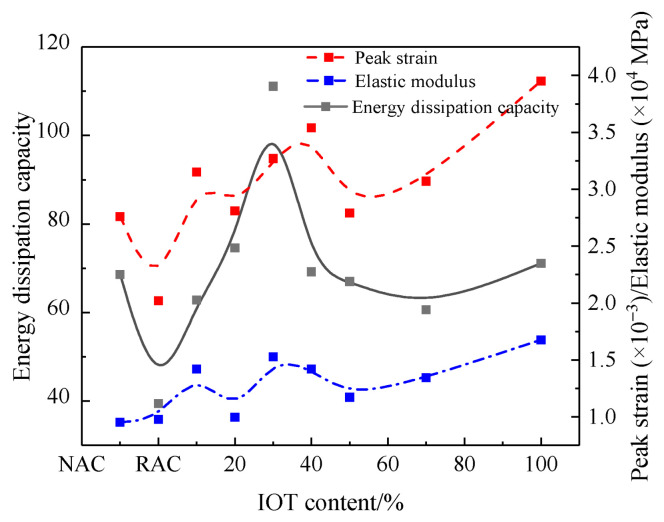
Peak strain, elastic modulus and energy dissipation capacity of the different concretes.

The peak strain of concrete is shown in Figure 6. It can be seen that the peak strain of RAC was lower than that of NAC. This was consistent with the results of Kazmi et al. [31] and Chen et al. [32]. However, in some previous studies, the incorporation of RCA resulted in an increase in the peak strain in concrete [33,34]. But, in this study, this behavior was not observed, which might be attributed to the elastic modulus of concrete. IOTs can result in irregular changes in the peak strain in RAC. This fluctuation can be attributed to the non-uniform dispersion of IOTs in concrete, resulting in a non-uniform concrete matrix. It was worth noting that the peak strain in all of the IOT groups was superior to that in NAC and RAC. For example, the peak strains of RAC-IOT10, RAC-IOT20, RAC-IOT30, RAC-IOT40, RAC-IOT50, RAC-IOT70, and RAC-IOT100 were 55.9%, 39.0%, 62.1%, 75.0%, 38.0%, 51.8%, and 95.5% higher than those of RAC, and 14.1%, 1.7%, 18.6%, 28.1%, 1.0%, 11.1%, and 43.1% higher than those of NAC, respectively.

#### 4.1.3. Elastic Modulus

The elastic modulus of the tested concretes is shown in Figure 6. Similar to the peak strain results, the elastic modulus also showed a large fluctuation. This result was also observed by Thomas et al. [35]. In general, the elastic modulus of all concrete containing IOTs was higher than that of RAC and NAC. For example, the elastic modulus of RAC-IOT10, RAC-IOT20, RAC-IOT30, RAC-IOT40, RAC-IOT50, RAC-IOT70, and RAC-IOT100 was 45.2%, 1.8%, 56.0%, 45.2%, 19.8%, 37.4%, and 71.4% higher than that of RAC, respectively. Obviously, the incorporation of IOTs into concrete will lead to the increase in elastic modulus. This may be due to the bonding between aggregate and cement [27].

#### 4.1.4. Energy Dissipation Capacity

The energy dissipation capacity of concrete was determined by calculating the area under the stress–strain curve using MATLAB 2021 software. The energy dissipation capacity directly reflected the concrete’s ability to resist brittle failure under compression. The stronger the energy dissipation capacity, the higher the safety of concrete under external load.

The calculation formulas are listed below:

W=∫σdε

where *W* is the energy dissipation capacity, *ε* is strain, and *σ* is stress.

The energy dissipation capacity of concrete is shown in Figure 6. RAC had the lowest energy consumption capacity. The energy dissipation capacity of RAC can be improved by adding IOTs. When the content of IOTs was less than 30%, the energy dissipation capacity increased with the increase in the IOT content. Meanwhile, when the content of IOTs exceeded 30%, the opposite trend was observed.

The area under the stress–strain curve was a measure of material toughness. Fanella et al. [36] defined the toughness of concrete as the ratio of the reinforced matrix to unreinforced matrix (control). Herein, the ratio of all TRACs was greater than 1. For example, the energy dissipation capacity of RAC-IOT10, RAC-IOT20, RAC-IOT30, RAC-IOT40, RAC-IOT50, RAC-IOT70, and RAC-IOT100 was 59.4%, 89.3%, 182.0%, 75.6%, 70.1%, 53.8%, and 80.5% higher than that of RAC, respectively. RAC-IOT30 showed the highest energy dissipation capacity, followed by RAC-IOT20. Among them, the energy dissipation capacity of RAC-IOT20 and RAC-IOT30 was 8.7% and 62.0% higher than that of NAC, respectively. The addition of an appropriate amount of concrete can make the concrete have a partial buffer in the process of energy dissipation, which can ensure that the concrete shows obvious warning signs in the overload failure stage. As will be discussed in Section 4.3, the presence of IOTs incorporated into the concrete impacted the porosity. The porosity of the concrete decreased at first and then increased with the increase in IOT content, and the decrease in porosity thickened the pore wall surface [37]. Therefore, the energy dissipated by generating and spreading microcracks inside the concrete increased, resulting in an increase in the energy dissipation capacity.

### 4.2. SEM Analysis

**Figure 7:**
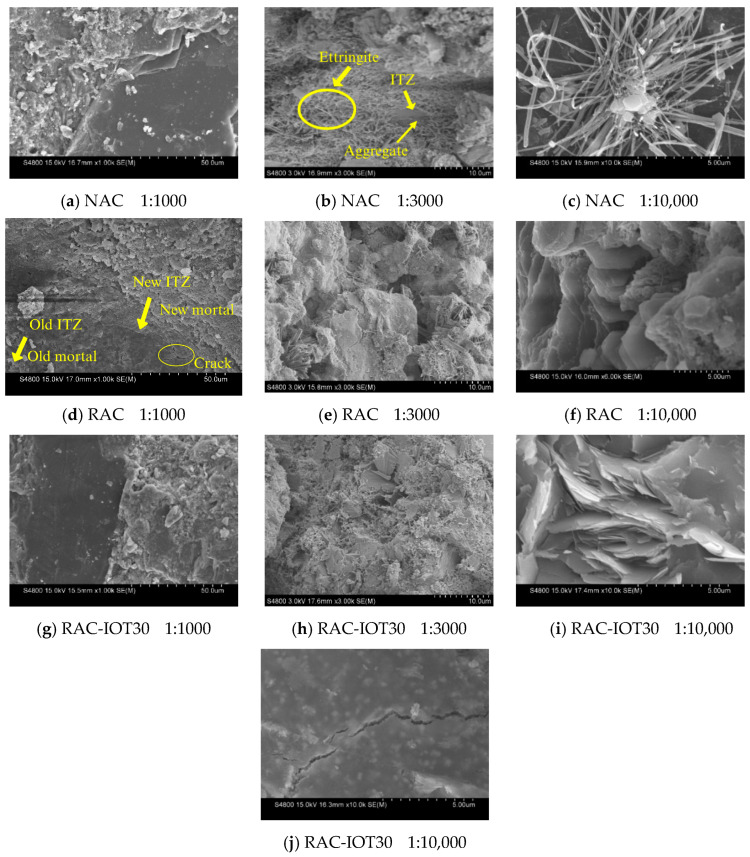
SEM images of NAC, RAC, and RAC-IOT30.

**Figure 8:**
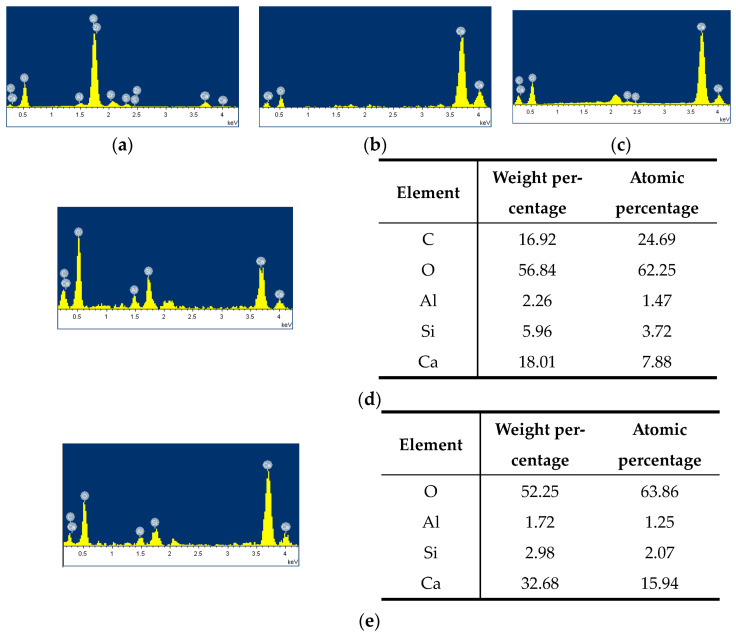
Energy spectrum analysis of hydration products: (**a**) thin needle-shaped crystal; (**b**) hexagonal flake crystal; (**c**) columnar crystal; (**d**) old ITZ; (**e**) new ITZ.

In order to understand the relationship between the properties, composition, and microstructure of NAC, RAC, and TRAC, an SEM was used to characterize the microstructure and hydration product features. At least ten microscopic images were taken per specimen, and representative images are shown in Figure 7. The SEM images of NAC cured for 28 days are presented in Figure 7a–c. It is well known that the ITZ is a weak area in concrete with a loose structure and rich pores, which had a large influence on the mechanical properties of concrete [38,39]. Figure 7a reflects the interface morphology of the cement matrix and NCA in NAC. The interface was found to be tightly bound, and there were almost no cracks at the interface. Figure 7b illustrates the representative image of NAC magnified 3000 times. From the image, we can see that there existed a lot of thin needle-shaped crystals in the cement matrix, and the bond between the sand and cement matrix was tight. Figure 7c shows the SEM image of thin needle-shaped crystals under 10,000× magnification. The element analysis of the thin needle-shaped crystals was performed using EDS, and the corresponding results are shown in Figure 8a. The main elements detected were O, Al, Si, S, and Ca, indicating that the thin needle-shaped crystals were ettringite (3CaO·Al_2_O_3_·3CaSO_4_·32H_2_O). Ettringite can effectively enhance the cohesive force between the cement matrix and aggregate [40]. Moreover, in Figure 7b, a few petal-shaped reticulated calcium silicate hydrates (C-S-H) are clearly visible. C-S-H gel can bond the intergranular components together and had a beneficial effect on the strength of the cement matrix.

Figure 7d–f show the SEM images of RAC with different magnifications. In Figure 7d, it can be seen that RAC has a slightly loose microstructure and uneven pore distribution compared with NAC. Some cracks were observed on the surface of RCA, which were not observed on the surface of the coarse aggregate of NAC. The old ITZ between the original aggregate and the old mortar, and the new ITZ between RCA and the new mortar can be seen in Figure 7d. The microstructure of the ITZ of the concrete largely determined the macroscopic performance of the concrete. Figure 8d,e show the EDS analysis results of the old ITZ and the new ITZ of RCA at 28 days of age. As can be seen from the figure, the weight percentages of SiO_2_, Al_2_O_3_, and CaO in the new ITZ were much higher than those in the old ITZ. This indicated that the content of C-S-H in the new ITZ was higher than that in the old ITZ, explaining the strength reduction caused by the use of RCA in concrete in Section 4.1.1. Figure 7e shows the representative image of RAC under 3000× magnification, from which columnar crystals are observed. The corresponding EDS analysis results are shown in Figure 8c. The main elements of the columnar crystals were S, Ca, and O, indicating that the columnar crystals were gypsum. A large number of hexagonal flake crystals (magnification 10,000×) can also be observed in Figure 7f. EDS analysis of the crystal (Figure 8b) showed that the main elements detected were O and Ca, indicating that the hexagonal flake crystals were Ca(OH)_2_. Ca(OH)_2_ crystals had a low strength and were easy to break under the action of an external force, which was also the reason for the decrease in the macroscopic mechanical properties of concrete containing RCA.

Figure 7g–j show the SEM image of RAC-IOT30. It can be observed from Figure 7g that the IOTs were well bonded to the surrounding cement matrix, and the microstructure was dense. A large number of C-S-H gels, thin needle-shaped crystals (ettringite), and IOTs can be found in Figure 7h. Although the IOT surface was at an angle to the matrix, a dense ITZ around it can still be observed. In addition, the three-dimensional grid-like C-S-H gel content observed in RAC-IOT30 was significantly higher than that in RAC and NAC. This showed that IOTs had pozzolanic activity, and can react with Ca(OH)_2_ to form C-S-H gel, which improved the dense microstructure and strength characteristics of concrete. This result was consistent with the results in Section 4.1.1 demonstrating that adding IOTs into RAC can improve the axial compressive strength of concrete. Similar conclusions have been reported by previous studies [41]. A large number of rose-petal-like single-sulfur calcium sulfoaluminate hydrate (AFm) molecules were distributed at the junction of the IOTs and cement matrix (Figure 7i). When the gypsum content was insufficient, ettringite were transformed into AFm, which had a positive impact on the strength.

In addition, a few cracks with a width of about 0.2 μm were observed on the surface of the IOT particles (Figure 7j). The higher the content of IOTs, the more cracks in the aggregates, and the lower the mechanical properties of concrete. Therefore, the amount of IOTs must be strictly controlled to avoid excessive performance degradation. This result showed that the mechanical properties were closely related to the microstructure characteristics.

### 4.3. Pore Structure

**Figure 9:**
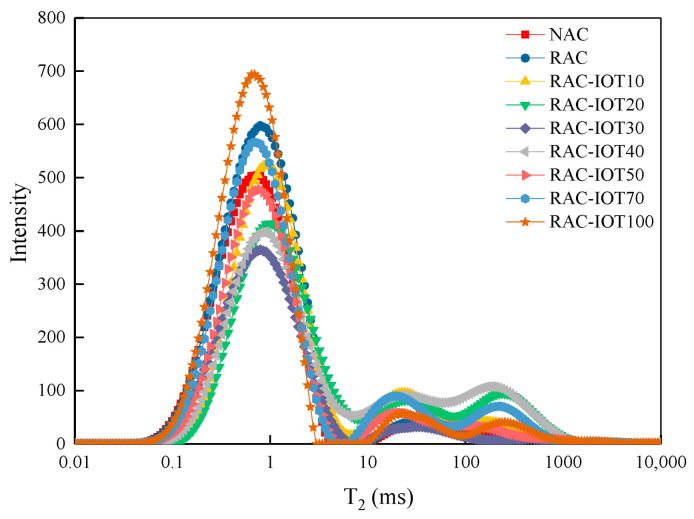
T_2_ spectrum of the tested concrete.

Typically, the abscissa of the T_2_ spectrum distribution curve was related to the pore size in a porous medium. The larger the pore size, the greater the degree of water freedom in the pore, and the longer the relaxation time. The ordinate represented the signal intensity, and the signal intensity reflected the number of pores. The higher the amplitude, the greater the signal intensity of water in the pores of the sample and the pore number corresponding to the radius. NMR tests were performed on different concrete specimens in this study, and the T_2_ spectrum curve is shown in Figure 9. In this study, the T_2_ spectrum curves of different concrete specimens were multi-peak curves. Three peaks were observed for all specimens. The integral area of the T_2_ spectrum curve indicated the amount of total pore water in the concrete. Therefore, NMR techniques can be used to study the porosity, pore size, and pore number of porous materials.

It can be seen from Figure 9 that the pore structure of concrete has changed significantly due to different material content. On the whole, the addition of IOTs caused the pore size distribution of concrete to change greatly. For example, when the IOT content was 40%, the area under the curve of the main signal peak (the first peak) was just 69.031% of the total area of the curve, indicating that the pores with a size not within this range still accounted for a large proportion. Besides the main signal peak, there were also two larger minor signal peaks, which indicated that there were pores with a larger diameter in the concrete. For concrete containing 30% IOTs (RAC-IOT30), the area under the curve of the main signal peak was 91.708% of the total area of the curve, which was much larger than the area under the curve of the minor signal peak. This result indicated that the pores in RAC-IOT30 were mainly micropores, and the addition of an appropriate amount of IOTs can optimize the pore size distribution. The ordinate represented the pore number. It can be seen from the amplitude of the main signal peak that with the increase in IOT content, the amplitude first decreased and then increased. When the IOT content was 30%, the amplitude of the main signal peak decreased by 39.05% compared with the concrete containing 0% IOTs. When the IOT content increased to 100%, the amplitude of the main signal peak increased by 15.80% and 89.99%, respectively, compared with the concrete containing 0% and 30% IOTs. Overall, the addition of an appropriate amount of IOTs greatly reduced the number of pores, but when excessive IOTs were added, the number of pores increased significantly.

Porosity is a parameter that determines the degree to which the infiltration of solution can easily occur within tissues. The saturated water content per unit volume of sample was proportional to its porosity. Therefore, the nuclear magnetic signal per unit volume was directly proportional to the porosity. When other factors were not considered, this porosity was the total porosity [42], and the porosity of different concrete specimens can be obtained, as shown in Table 6. RAC had higher porosity than NAC. An appropriate amount of IOTs added to the concrete was beneficial to the reduction in porosity. For example, the porosity of RAC-IOT10, RAC-IOT20, RAC-IOT30, RAC-IOT40, RAC-IOT50, and RAC-IOT70 was 49.0%, 23.7%, 35.6%, 26.7%, 28.4%, and 14.8% lower than that of RAC, respectively. It can be seen that the incorporation of 30% IOTs can achieve the lowest porosity compared to other substitution rates. This was consistent with the research results that concrete containing 30% IOTs had the best macroscopic compressive strength. The reason was that the irregular angular shape of IOTs increased its interlock with the cement matrix. At the same time, the low pozzolanic activity of IOTs increased the content of C-S-H gel in the concrete, improved the density of the matrix, and enhanced the strength development of the matrix.

## 5. Conclusions

By analyzing the macroscopic mechanical properties and microstructure of NAC, RAC, and TRAC, the following conclusions can be drawn:

(1) The axial compressive strength and energy dissipation capacity of concrete first increased and then decreased when the IOT incorporation ratio continued to increase. The concrete containing 30% IOTs exhibited the highest axial compressive strength and energy dissipation capacity.
(2) The peak strain and elastic modulus in all of the IOT groups were superior to those in NAC and RAC. The mechanical test shows that TRAC’s mechanical properties were the best when the IOT content was 30%, on the whole.
(3) The SEM analysis revealed that RAC had a slightly loose microstructure distribution compared with NAC. Some cracks were observed on the surface of RCA and a large number of hexagonal flake Ca(OH)_2_ crystals were found in the matrix. The EDS analysis showed that the content of C-S-H gel in the new ITZ was higher than that in the old ITZ. The content of C-S-H gel was much higher in TRAC than in RAC and NAC, and the microstructure of TRAC was denser and uniform. A large number of rose-petal-like AFm molecules were observed at the junction of the IOTs and cement matrix. This SEM observation was consistent with the mechanical properties. A few cracks with a width of about 0.2 μm were observed on the surface of IOT particles. This might be the reason that excessive IOTs weakened the strength of concrete. Meanwhile, the hardness of IOTs was lower than that of silica sand. Therefore, the amount of IOTs must be controlled.
(4) IOTs can effectively reduce the porosity, pore size, and pore number of concrete, the porosity was the smallest when the content of IOTs was 30%. However, when excessive IOTs were added, the number of pores increased significantly.

From the macroscopic and microscopic analysis, it can be determined that the right amount of IOTs played the role of filling the pores and promoting the hydration of the cementite material, improving the compactness of the concrete, and thus improving its mechanical properties. Overall, the optimal usage was 20% to 40% to fully exploit the resource value and obtain the most favorable results.

However, a lot of research is still needed for the application of IOTs in engineering. Future research will focus on the enhancement effect of IOTs with different fineness parameters on RAC. In addition, the effect of sulfur content on IOTs should be studied.

## Figures and Tables

**Table 1:** Chemical elements of cement and IOTs.

Mineral Composition	C	O	Mg	Al	Si	S	K	Ca	Fe	Ti
Cement	4.12	47.54	0.74	1.54	6.11	1.10	0.53	56.84	0.93	0.01
IOTs	0	56.49	6.95	8.38	17.46	-	3.02	1.68	5.28	0.74

**Table 2:** Main performance indicators of cement.

Standard Consistency Water Consumption (%)	Initial Setting Time (min)	Final Setting Times (min)	Fineness (45 μm)	Flexural Strength (MPa)		Compressive Strength (MPa)	
				3d	28d	3d	28d
28	160	280	2.8	5.2	6.8	19	42.2

**Table 3:** Main performance indicators of aggregates.

Performance Indicator	Apparent Density (kg/m^3^)	Bulk Density (kg/m^3^)	Crush Value (%)	Water Absorptivity (%)	Mud Content (%)	Moisture Content (%)	Organic Matter Content	Alkali Aggregate Reaction
NCA	2941	1749	10.3	1.33	0.72	0.8	Qualified	Qualified
RCA	2536	1467	14.8	7	1.86	3.02	Qualified	Qualified
Norm values of NCA	≥2500	≥1300	≤16	-	≤1.0	-	Qualified	Qualified
Silica sand	2764	1830	12	2.12	1.2	4.1	Qualified	Qualified
IOTs	2745	1824	19.53	8.7	2.9	1.45	Qualified	Qualified
Norm values of silica sand	-	-	≤10	-	≤3.0	-	Qualified	Qualified

Remarks: the norm values referred to the Chinese national standard [21].

**Table 4:** Main physical property indexes of water-reducing agent.

Density (g/m^3^)	pH Values	Water Solubility	CL Content (%)	Na_2_SO_4_ Content (%)	R_2_O Content (%)
1.05 ± 0.2	6~7	Intermiscible	≤1.0	≤2.0	≤5.0

**Table 5:** Mix proportions.

Specimen	Water (kg/m^3^)	Cement (kg/m^3^)	NCA (kg/m^3^)	RCA (kg/m^3^)	Silica Sand (kg/m^3^)	IOTs (kg/m^3^)
NAC	215	537.5	1062.9	0	572.3	0
RAC	215	537.5	735.3	315.1	565.6	0
RAC-IOT10	215	537.5	739.4	316.9	511.9	56.9
RAC-IOT20	215	537.5	743.5	318.6	457.5	114.4
RAC-IOT30	215	537.5	747.4	320.3	402.5	172.5
RAC-IOT40	215	537.5	751.3	322.0	346.8	231.2
RAC-IOT50	215	537.5	755.2	323.6	290.4	290.4
RAC-IOT70	215	537.5	762.6	326.8	176.0	410.6
RAC-IOT100	215	537.5	773.3	331.4	0	594.8

**Table 6:** Porosity of the tested concrete.

Specimen	Porosity (%)	Specimen	Porosity (%)
NAC	11.89	RAC	14.63
RAC-IOT10	13.32	RAC-IOT20	11.17
RAC-IOT30	9.42	RAC-IOT40	10.72
RAC-IOT50	10.48	RAC-IOT70	12.46
RAC-IOT100	15.67		

## Data Availability

Data are contained within this article.

## Abbreviations

TRAC	Recycled aggregate concrete containing iron ore tailings
IOTs	Iron ore tailings
NCA	Natural coarse aggregate
NAC	Natural aggregate concrete
RCA	Recycled coarse aggregate
RAC	Recycled aggregate concrete
ITZ	Interfacial transition zone
SEM	Scanning electron microscope
C-S-H	Calcium silicate hydrate
AFm	Monosulfate calcium sulfoaluminate hydrate
NMR	Nuclear magnetic resonance
